# Changes in the methylation status of the *Oct3/4, Nanog,* and *Sox2* promoters in stem cells during regeneration of rat tracheal epithelium after injury

**DOI:** 10.18632/oncotarget.13818

**Published:** 2016-12-07

**Authors:** Ying Zhou, Nan Song, Xin Li, Ying Han, Zihan Ren, Jing-xian Xu, Yu-chen Han, Fang Li, Xinshan Jia

**Affiliations:** ^1^ Department of Pathology, College of Basic Medical Sciences, China Medical University, Shenyang, 110001, China; ^2^ Department of Pathology, First Affiliated Hospital of China Medical University, Shenyang, 110001, China; ^3^ Department of Emergency, First Affiliated Hospital of China Medical University, Shenyang, 110001, China; ^4^ Department of Physiology, College of Life Science and Biopharmaceutics of Shenyang Pharmaceutical University, Shenyang, 110016, China; ^5^ Department of Pathology, Shenyang Medical College, Shenyang, 110001, China; ^6^ Department of Ophthalmology, The 4th Affiliated Hospital, Eye Institute, China Medical University, The Key Laboratory of Lens Research, Shenyang 110005, China; ^7^ IVF Michigan, Bloomfield Hills, MI, 48304, USA

**Keywords:** tracheal epithelium, stem cell, Oct3/4, methylation, silent gene

## Abstract

We investigated the relationship between promoter methylation and tracheal stem cell activation. We developed a model of rat tracheal epithelium regeneration after 5-fluorouracil (5-FU)-induced injury. Using immunohistochemistry and Western blotting, the expression levels of the stem cell pluripotency regulator Oct3/4 and differentiation marker CK14 were measured after 5-FU treatment. The methylation status of the *Oct3/4*, *Nanog*, and *Sox2* promoters was investigated using methylation-specific PCR. Additionally, the effects of 5-azacytidine (5-azaC), a demethylating agent, on *Oct3/4*, *Nanog*, and *Sox2* mRNA and protein expression were evaluated. Finally, we measured the activity of the maintenance and *de novo* DNA methyltransferases DNMT1, DNMT3a, and DNMT3b. Our data indicate that *Oct3/4*, *Sox2*, and *Nanog* are transiently expressed in response to 5-FU-induced injury, and then they are gradually silenced as the cells differentiate. DNA methylation can result in silencing of gene expression, and it can determine whether tracheal stem cells are in an active or dormant state. Treatment with 5-FU reversed the methylation of the *Oct3/4*, *Nanog*, and *Sox2* promoters, which corresponded to increases in Oct3/4, Nanog, and Sox2 mRNA and protein. Thus, both maintenance and *de novo* methyltransferases are involved in regulating tracheal stem cell dormancy and activation.

## INTRODUCTION

Stem cells in adult animals normally exist in a state of dormancy in which they remain in the G0 phase of the cell cycle and do not undergo mitotic division [1–3]. However, under specific conditions or in response to various factors *in vivo*, stem cells can be activated, re-enter the cell cycle, and ultimately differentiate into specific tissues [4–6]. These processes can occur during tissue regeneration in response to injury and other factors. An understanding of the mechanisms responsible for reactivation of dormant stem cells is important in order to modulate stem cell behavior and develop stem cell-based therapies for disease [7–9].

The transcriptional regulator Oct3/4 is expressed in both pluripotent embryonic stem (ES) cells and germline cells. Inactivation of Oct3/4 results in the loss of pluripotency and apoptosis [10–13]. Oct3/4 is one of a small group of pluripotency regulators that includes Nanog and Sox2, which reinforce each other's expression [14–19]. In this study, we investigated the roles of both tracheal stem cells and basal cells during the regeneration of the rat tracheal epithelium after injury. The relationship between *Oct3/4*, *Nanog*, and *Sox2* promoter methylation and tracheal stem cell activation was analyzed using methylation-specific PCR (MSPCR). We used a previously developed *ex vivo* model of rat tracheal epithelium regeneration after 5-fluorouracil (5-FU)-induced injury. In addition, rat tracheal stem cells were treated with the demethylating agent 5-azacytidine (5-azaC), and changes in the expression of *Oct3/4*, *Nanog*, and *Sox2* were quantified. Our results demonstrate that tracheal stem cell activation is controlled by epigenetic modifications.

## RESULTS

### Oct3/4 is transiently expressed in the rat tracheal epithelium after 5-FU-mediated injury

**Figure 1:**
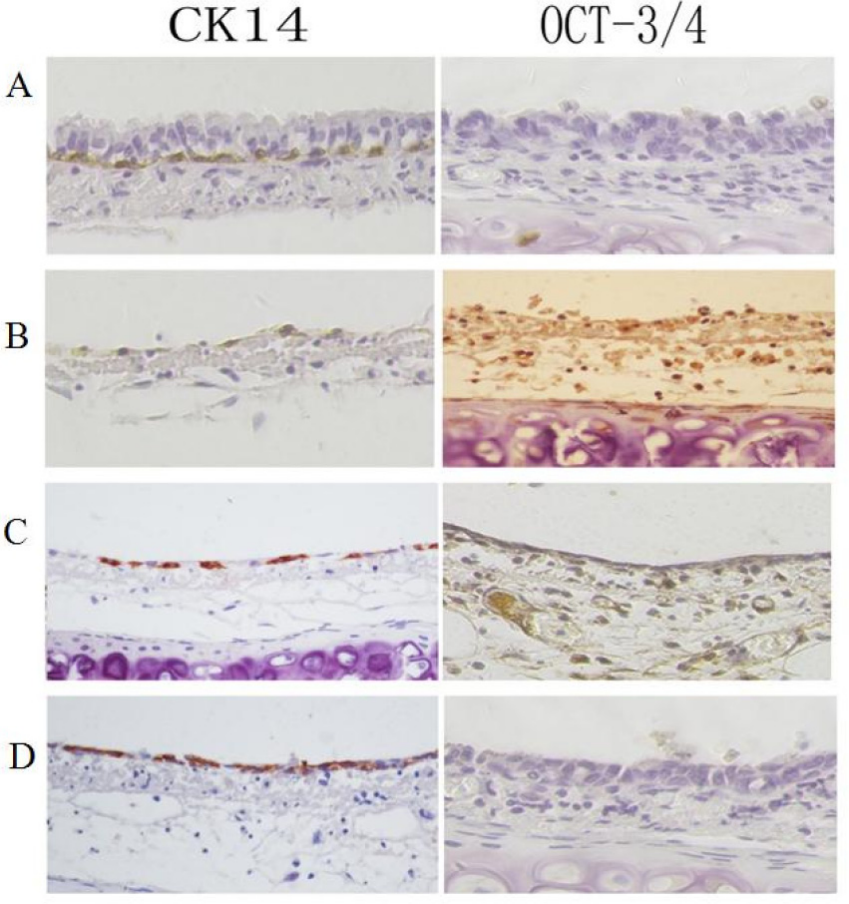
Immunohistochemical analysis of Oct3/4 and CK14 expression during rat tracheal epithelium recovery after 5-FU-induced injury. Top-bottom (**A**) Oct3/4 is not detected in untreated rat tracheal epithelial cells. Positive CK14 staining is observed in basal cells. (**B**) Oct3/4-positive cells observed after treatment with 5-FU. CK14-positive cells are not observed on the basement membrane. (**C**) Increased numbers of Oct3/4-positive cells and the appearance of CK14-positive cells. (**D**) Increased numbers of CK14-positive cells and a corresponding decrease in the number of Oct3/4-positive cells. The expression of Oct3/4 and CK14 reached near-normal levels after 48 h.

We investigated the expression of Oct3/4 in the rat tracheal epithelium after 5-FU-mediated injury by immunohistochemistry. Oct3/4 expression was not observed in the normal rat tracheal epithelium. Immediately after 5-FU treatment, there were only a few cells in G0 (reduced cytoplasm) that were attached to the basement membrane. These cells were Oct3/4-positive. As the tracheal epithelium recovered, the number of Oct3/4-positive cells gradually increased to a maximum. At this point, Oct3/4 levels began to decrease, and they returned to baseline by 48 h. CK14 expression (a marker of differentiation) was observed in the cytoplasm of basal cells in the normal tracheal epithelium. However, 5-FU treatment resulted in a transient decrease in CK14 expression. This decrease was followed by a gradual increase over time to nearly normal levels at 48 h (Figure 1).

### Transient expression of Oct3/4 in the tracheal epithelium detected by western blotting

**Figure 2:**
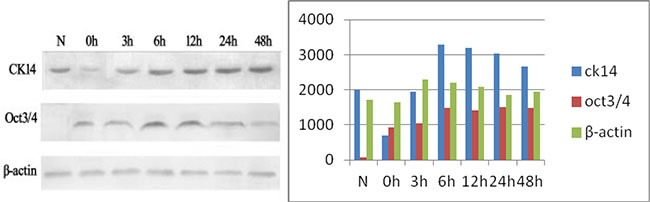
Oct3/4 and CK14 expression in the tracheal epithelium during recovery after 5-FU-induced injury N. Extracts from normal tracheal epithelial cells showing high CK14 expression and no detectable Oct3/4 expression; 0 h: Low CK14 expression after treatment with 5-FU. Minimal Oct3/4 expression is observed at this time-point; 3–6 h: An increase in both Oct3/4 and CK14 expression is observed, with maximal expression at 6 h; 12–48 h: CK14 expression is restored to normal levels by 48 h, while Oct3/4 expression gradually decreases.

Consistent with the immunohistochemical data, Oct3/4 expression was not detectable in the normal rat tracheal epithelium. After treatment with 5-FU, Oct3/4 expression increased and reached a maximal level at approximately 6 h. The expression subsequently decreased to relatively low levels after approximately 48 h. CK14 was highly expressed in normal rat tracheal epithelium. Immediately after treatment with 5-FU, only trace amounts of CK14 were detected. CK14 expression increased gradually over time and returned to approximately normal levels after 48 h (Figure 2).

### 5-FU induces demethylation of the Oct3/4, Nanog, and Sox2 promoters in rat tracheal epithelial cells

**Figure 3:**
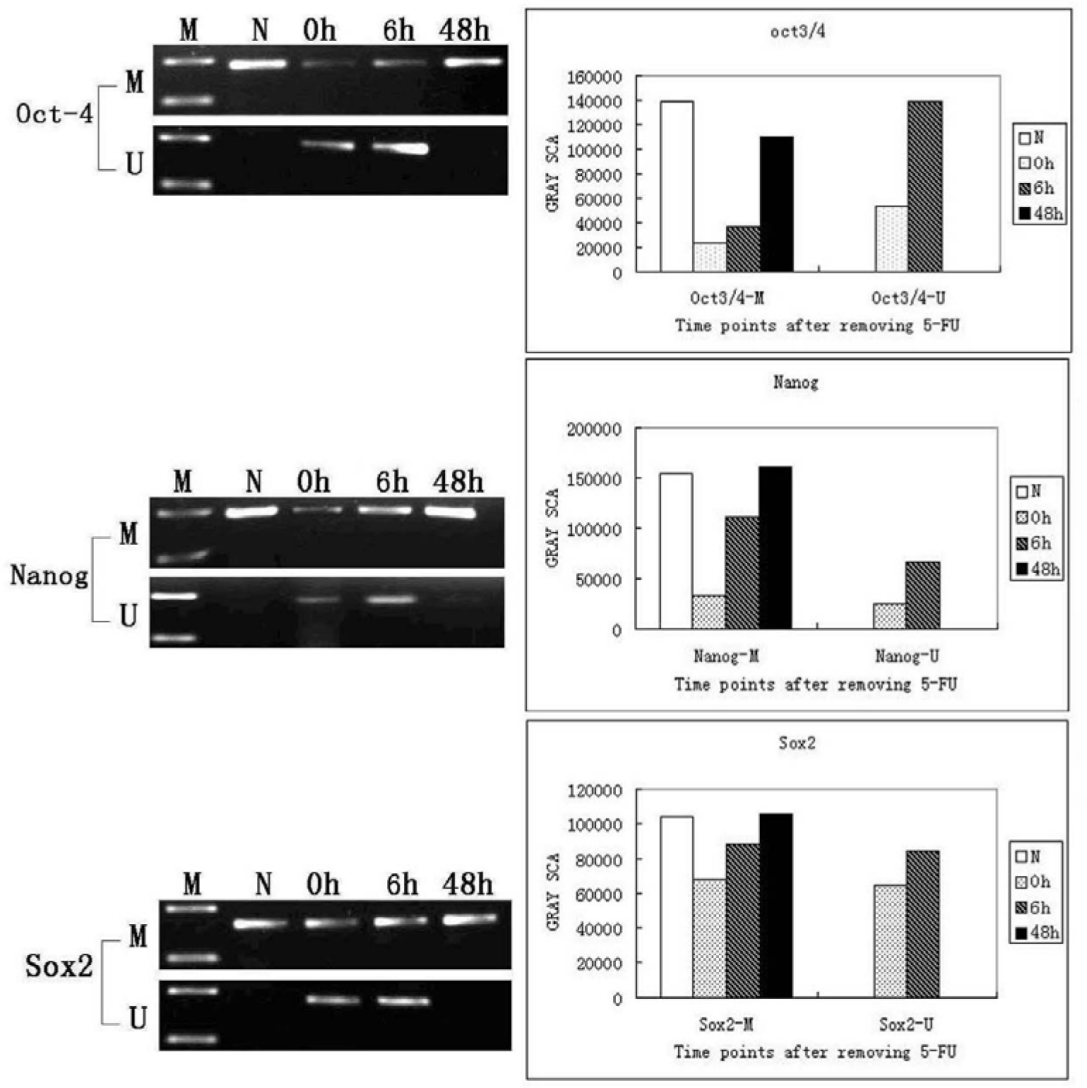
MSPCR analysis of the methylation status of the *Oct3/4*, *Nanog*, and *Sox2* promoters in normal and 5-FU-treated tracheas N: Normal tracheal epithelial cells. Amplification of the methylated alleles of the *Oct3/4*, *Nanog*, and *Sox2* genes is detected; 0 h: Immediately after removal of 5-FU, amplification of the unmethylated alleles of all three genes is detected; 6 h: Amplification of the unmethylated alleles of all three genes is apparent 6 h after the removal of 5-FU; 48 h: Only the methylated alleles of the *Oct3/4*, *Nanog*, and *Sox2* genes are detected 48 h after removal of the 5-FU.

We next investigated the methylation status of the *Oct3/4*, *Nanog*, and *Sox2* promoters using MSPCR. The promoter regions of all three genes were methylated in normal tracheal epithelial cells (only methylated alleles were amplified). Both methylated and unmethylated alleles were detected in tracheal epithelial cells 0 h and 6 h after treatment with 5-FU (Figure 3). A significant increase in the methylated alleles was detected between 6 h and 48 h after 5-FU treatment, and only methylated alleles were detected in tracheal epithelial cells at 48 h. These results demonstrate that the promoter regions of these three genes are methylated in normal tissue, and that they are transiently demethylated in response to 5-FU-induced injury. The promoters gradually become remethylated as the epithelium is regenerated (Figure 3).

### Analysis of DNA methyltransferase activity in rat tracheal epithelial cells

**Figure 4:**
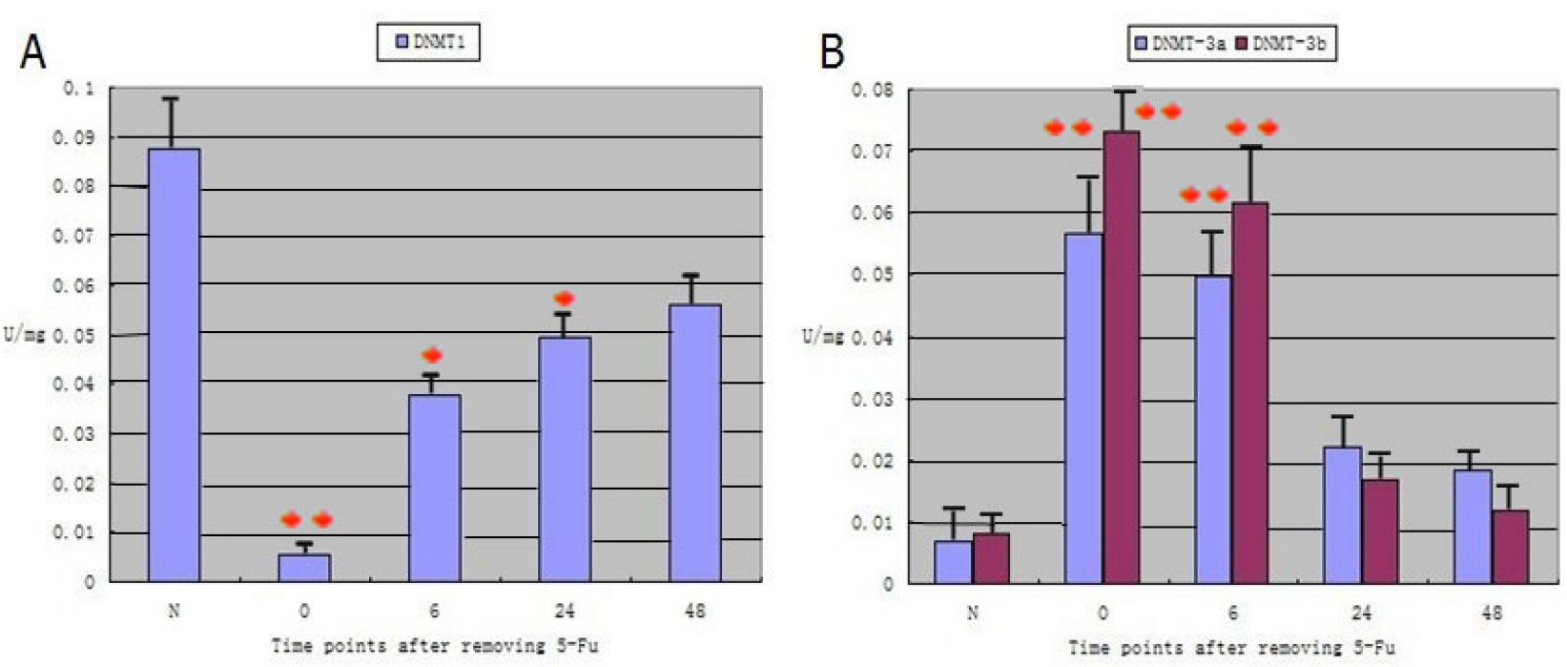
Analysis of DNMT1, DNMT3a, and DNMT3b activity in rat tracheal epithelial cells (*P* < 0.05) (**A**) Higher DNMT1 activity is observed in normal rat tracheal epithelial cells (N). A decreased in DNMT1 activity is observed immediately after treatment with 5-FU (0 h). A gradual increase in DNMT1 activity to near-normal levels is apparent between 0 h and 48 h, which is correlated with cellular differentiation. (**B**) Relatively low DNMT3a and DNMT3b activity is observed in normal tracheal epithelial cells. A significant increase in activity is observed 0 h after 5-Fu treatment. A gradual decrease in DNMT3a and DNMT3b activity to near-normal levels is observed between 0 h and 48 h, which is correlated with cellular differentiation.

In the normal rat tracheal epithelium, the activity of DNMT1, a maintenance DNA methyltransferase, was 0.0739 units/mg. DNMT1 activity significantly decreased after 5-FU treatment to 0.0010 units/mg, and then gradually increased as the cells differentiated to 0.0598 units/mg at 48 h (approximately the same level as in normal cells) (Figure 4). Relatively low activity of the *de novo* DNA methyltransferases, DNMT3a and DNMT3b, was observed in the normal tracheal epithelium (0.0044 units/mg and 0.0079 units/mg, respectively). However, the activity of both enzymes significantly increased (0.0642 units/mg and 0.0577 units/mg, respectively) immediately after 5-FU treatment (0 h). As the cells differentiated, DNMT3a and DNMT3b activity gradually decreased to 0.0046 units/mg and 0.0191 unit/mg, respectively, at 48 h. These levels were similar to those of normal cells. Thus, significant differences were observed in DNA methyltransferase activity between normal tracheal epithelial cells and 5-FU-treated cells at each time point analyzed (Figure 4).

### The effects of 5-azaC treatment on the repair of the rat tracheal epithelium

**Figure 5:**
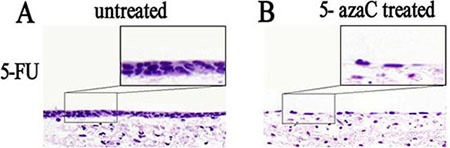
Analysis of the morphological effects of 5-azaC treatment on rat tracheal epithelium repair by HE staining (**A**) Double-layer appearance of the rat tracheal epithelium 30 h after 5-FU treatment. (**B**) Flat tracheal epithelial cells are observed in the 5-azaC-treated group.

We investigated the effects of 5-azaC treatment on the repair of the rat tracheal epithelium. Morphological changes in tracheal stem cells in the 5-FU untreated control) and 5-azaC-treated groups were evaluated in tissue specimens stained with hematoxylin and eosin (HE). In the untreated group, the tracheal epithelium had a double-layered appearance, while flat epithelial cells were observed in the 5-azaC-treated group (Figure 5). These data suggested that the tracheal epithelium was partly restored by 30 h in the control group. Stem cell proliferation was still observed at this time-point in the 5-azaC-treated group, which was indicative of a delay in differentiation. Thus, 5-azaC-induced demethylation could prolong the differentiation of tracheal epithelial cells.

### Enhanced expression of Oct3/4, Nanog, and Sox2 in tracheal epithelial cells after treatment with 5-azaC

**Figure 6:**
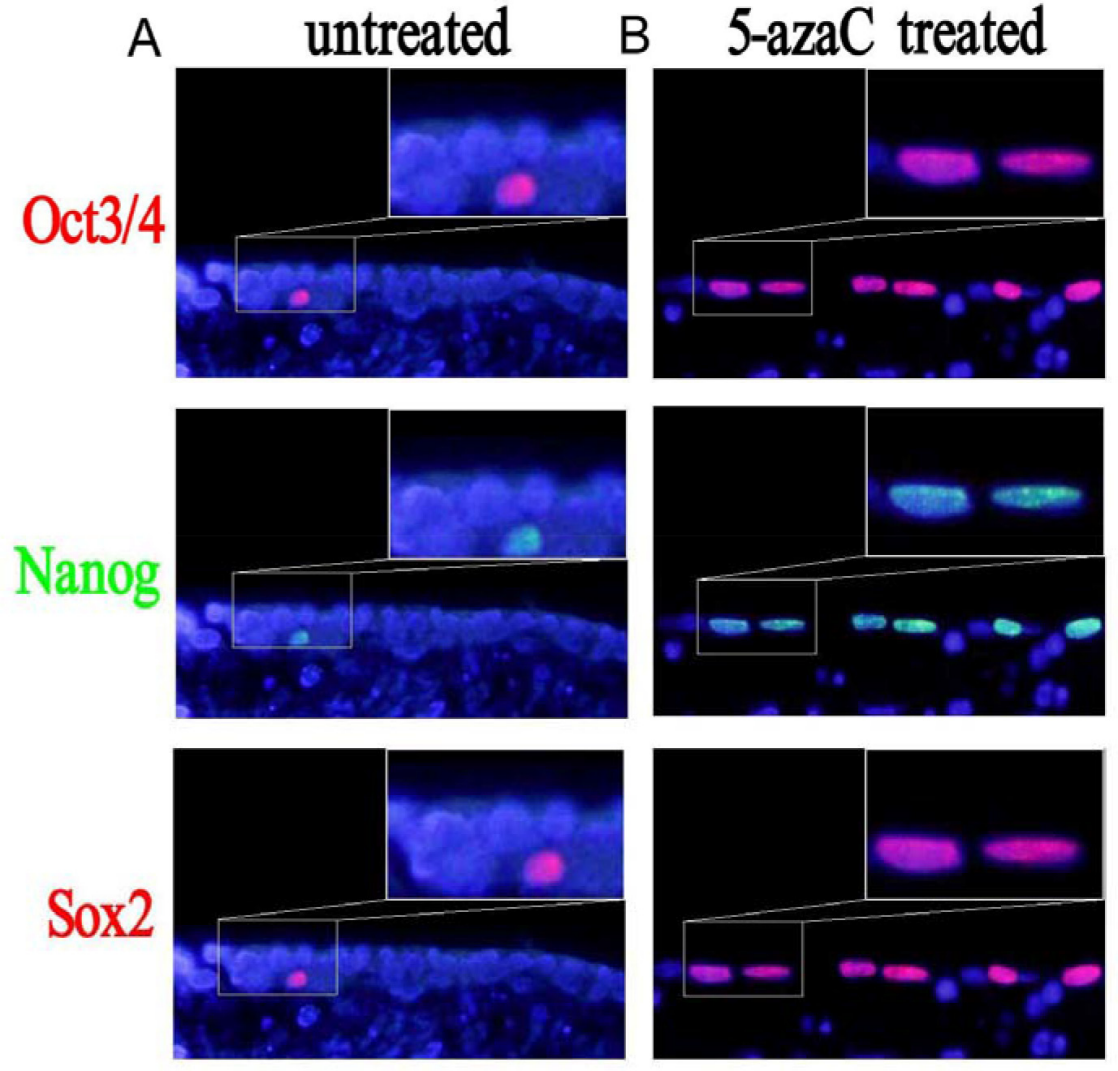
Changes in the expression of Sox2, Nanog, and Oct3/4 after 5-azaC treatment analyzed by immunofluorescence (**A**) In the 5-FU treated group, few Oct3/4-, Nanog-, and Sox2-positive cells are observed. (**B**) In the 5-azaC treated group, an increase in the number of Oct3/4-, Nanog-, and Sox2-positive cells is observed.

**Figure 7:**
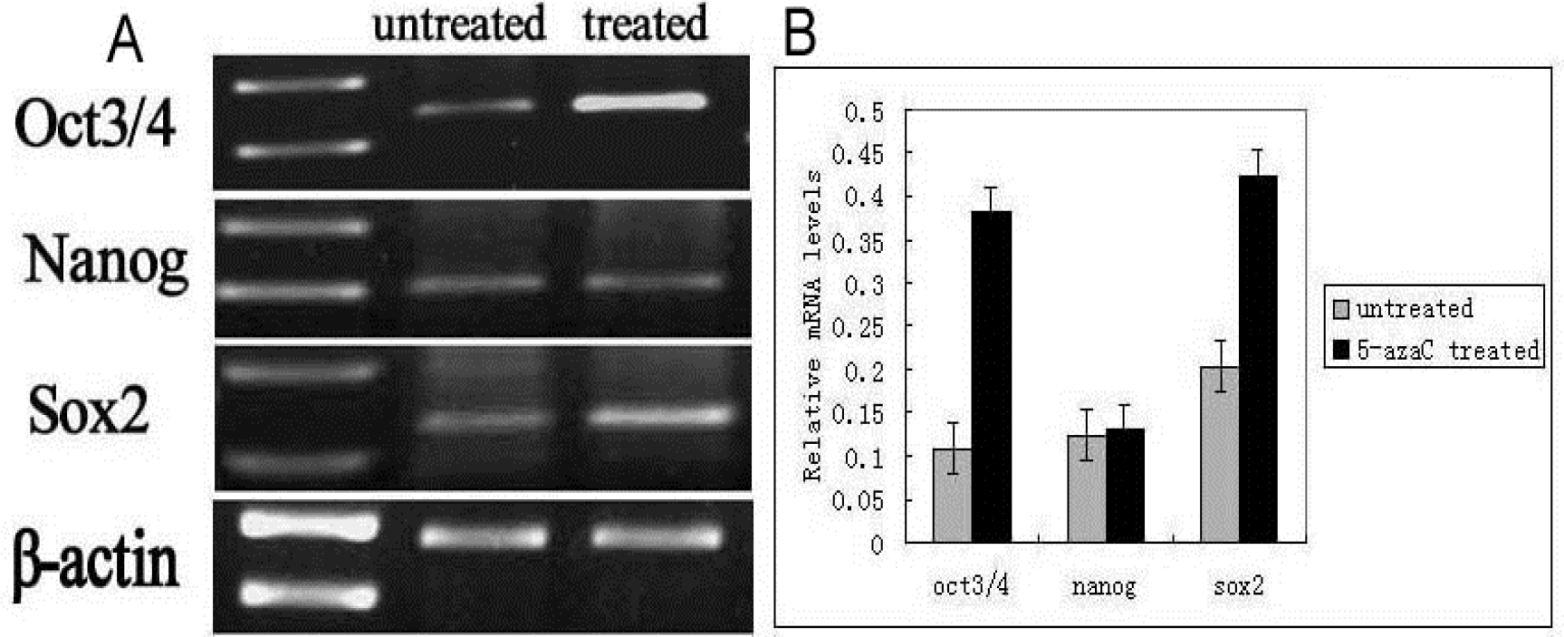
Quantification of the mRNA levels of *Oct3/4*, *Nanog*, and *Sox2* by RT-PCR (**A**) *Oct3/4*, *Nanog*, and *Sox2* mRNA levels in the rat tracheal epithelium in the 5-azaC-treated and untreated groups. (**B**) Relative mRNA expression of *Oct3/4*, *Nanog*, and *Sox2* in the rat tracheal epithelium in the 5-azaC- treated and untreated groups. Data are presented as the mean ± SD of three independent experiments.

We analyzed the expression of the Oct3/4, Nanog, and Sox2 proteins in tracheal epithelial cells after 5-azaC treatment by immunofluorescence (Figure 6). Interestingly, there were fewer Nanog-, Sox2- and Oct3/4-positive cells in the 5-FU control group compared to the 5-azaC-treated group. These results suggested that 5-azaC promoted the expression of *Oct3/4*, *Nanog*, and *Sox2* during repair of the tracheal epithelium after injury. We therefore examined the levels of *Oct3/4*, *Nanog*, and *Sox2* mRNA using RT-PCR (Figure 7). These data revealed higher *Oct3/4*, *Nanog* and *Sox2* expression in the 5-azaC-treated group compared to the 5-FU control group. Thus, demethylation of the *Oct 3/4*, *Nanog*, and *Sox2 by* 5-azaC enhance the expression of these genes in the tracheal epithelium.

## DISCUSSION

We analyzed the expression and methylation status of the *Oct3/4*, *Nanog*, and *Sox2* genes in rat tracheal epithelial cells following 5-FU induced injury. Our results indicate that these proteins can be used as tracheal stem cell markers. *CK14*, a marker of differentiated epithelial cells, was expressed in the basal cells of the normal tracheal epithelium, but the stem cell markers *Oct3/4*, *Nanog*, and *Sox2* were not expressed in the normal tracheal epithelium. Thus, stem cells in the normal epithelium are in a state of dormancy. Following 5-FU-induced injury of the tracheal epithelium, proliferating cells died, while CK14-negative and Oct3/4-positive cells in G0 phase remained on the basement membrane. The number of Oct3/4-positive cells transiently increased, whereas the number of CK14-positive cells transiently decreased. This suggests that Oct3/4-expressing tracheal stem cells were selectively activated in response to injury. They then differentiated into various cell types, including ciliated, basal cells (which normally express CK14) and secretory cells.

Previous studies have suggested that basal cells are actually tracheal stem cells [20, 21]. However, based on the following data, we have concluded that basal cells are distinct from tracheal stem cells. First, stem cells are undifferentiated cells that do not express differentiated cell markers. However, basal cells contain many cellular organelles and express various cytokeratins, suggesting that basal cells have differentiated. Second, the number of basal cells is not correlated with the number of stem cells in tissues. The number of stem cells in adult tissues is actually quite low. For example, hepatic stem cells account for only 0.003% of all liver cells, and hematopoietic stem cells represent only 0.001% of hematopoietic cells. In contrast, basal cells in the trachea represent approximately 30% of all tracheal epithelial cells, suggesting they are not stem cells. Hence, only Oct3/4-positive cells are tracheal stem cells [22–25].

Regular changes in the expression of the *Oct3/4*, *Nanog*, and *Sox2* genes (silent-active-silent) were observed during the process of tracheal epithelium repair after 5-FU-induced injury. Our data indicate the expression of these genes is epigenetically regulated by promoter methylation. We previously showed that *Oct3/4*, *Nanog*, and *Sox2* are not expressed in the normal tracheal epithelium [22, 23]. This is because the promoters of these genes are normally methylated and therefore transcriptionally silenced. After treatment with 5-FU, the G0 phase cells that remained on the basement membrane expressed *Oct3/4*, *Nanog*, and *Sox2*. At this time, the promoters were in a demethylated state, which allowed for gradual reactivation in response to injury. The number of cells in the unmethylated state increased as the cells proliferated. As the cells differentiated, the expression of *Oct3/4*, *Nanog*, and *Sox2* decreased, and the degree of methylation increased. *Oct3/4*, *Nanog*, and *Sox2* expression almost completely disappeared once repair was complete. We propose that the *Oct3/4*-, *Nanog*-, and *Sox2*-expressing cells are undifferentiated and multipotent tracheal stem cells.

We previously knew that DNA methylation can result in gene silencing, and that demethylation can lead to re-activation of gene expression, especially in embryonic stem cells [26–28]. Here, we demonstrated that the proliferation and differentiation of tracheal stem cells were correlated with promoter methylation state. We determined that 5-azaC-induced demethylation of the *Oct3/4*, *Nanog*, and *Sox2* promoters resulted in tracheal stem cell activation. In a study of non-tumor cells, methylation was the main cause of gene silencing [29]. We used the demethylating agent 5-azaC to study the effect of demethylation on gene expression in tracheal stem cells. Because 5-azaC has a certain degree of cellular toxicity, the tracheal epithelium completely differentiated became necrotic, and sloughed off after 5-azaC treatment. *Oct3/4*, *Nanog*, and *Sox2* were re-expressed in the differentiated tracheal epithelium. Some studies have found that 5-azaC reversed the differentiation ES cells and lead to *Oct3/4*, *Nanog*, and *Sox2* expression [30]. Our data support the hypothesis that 5-azaC-induced demethylation of the *Oct3/4*, *Nanog*, and *Sox2* activates gene expression. However, there are also other mechanisms that regulate stem cell proliferation and differentiation such as histone acetylation [31–33].

DNMT1 is generally thought to be the primary methyltransferase involved in the maintenance of methylation, whereas *de novo* methylation relies on the activities of DNMT3a and DNMT3b [34]. DNMT1 is the most abundant DNA methyltransferase and is mainly expressed during the S phase of the cell cycle. DNMT3a and DNMT3b directly interact in stem cells. During the differentiation of embryonic carcinoma cells and ES cells, these two enzymes cooperate to methylate the promoter regions of the *Oct3/4* and *Nanog* genes. Thus, removal of DNMT3b and DNMT3a can result in insufficient methylation and disordered *Oct3/4* expression [35–38].

Here, we demonstrated that in the normal tracheal epithelium, the activity of the maintenance methyltransferase DNMT1 increased and the activities of the *de novo* methyltransferases DNMT3a and DNMT3b decreased. *Oct3/4* expression was observed in G0 phase cells after 5-FU treatment. When the *Oct3/4* promoter was in the unmethylated state, the activity DNMT1 decreased while the activities of DNMT3a and DNMT3b increased. These results were indicative of reactivation of the normally silenced gene. During the differentiation of G0 phase cells, the expression of *Oct3/4* gradually disappeared. This was the result of methylation-induced gene silencing. During this process, DNMT1 activity increased, while the DNMT3a and DNMT3b activity returned to near-normal levels. Therefore, DNA methyltransferases function to both silence and activate the stem cell marker *Oct3/4*, leading to dormancy or activation of tracheal stem cells, respectively.

The results of our study question the traditional view that basal cells are stem cells. We propose that *Oct3/4*, *Nanog*, and *Sox2* are not only ES cell-specific markers, but also indicate, via their expression levels, the degree of cellular undifferentiation. Additional studies are required to elucidate the mechanisms responsible for the conversion of stem cells from dormancy to activation, which will be required for the development of stem cell-based therapies.

## MATERIALS AND METHODS

### Model for tracheal epithelial regeneration

This study was approved by the Institutional Review Board/Ethics Committee of the First Affiliated Hospital of China Medical University. Male and female Wistar rats (200 g each) were cared for in accordance with the guidelines of the Animal Care Committee of China Medical University. Animals were euthanized and tracheas excised under sterile conditions and then cultured in a 1:1 mixture of Dulbecco's Modified Eagle's Medium and Ham's F-12 medium (DMEM/F12) supplemented with 120 mg/mL 5-FU and 10% fetal bovine serum (FBS) for 12 h at 37°C. The 5-FU was then washed out and the tracheas cultured in DMEM/F12 media supplemented with 10% FBS for 0, 3, 6, 12, 24, or 48 h. The epithelial cells were then isolated from the tracheas for analysis.

Tracheal tissue was divided into a 5-FU group (no 5-azaC treatment) and a 5-azaC treatment group. The tissue was all incubated with 5-FU for 12 h, after which the medium was exchanged, and the tissue incubated for an additional 30 h. In the 5-FU group, the tracheal tissue was removed and placed in DMEM/F12 supplemented with 10% FBS for 30 h. The tracheal tissue was then stored until analysis. In the 5-azaC group, after treatment with 5-FU, the medium was exchanged and the tissue was incubated in DMEM/F12 supplemented with 10% FBS for an additional 24 h. The tissue was then removed and placed in DMEM/F12 containing 1 μm 5-azaC and incubated for 6 h. Following these incubations, the tracheal tissue was removed and stored until analysis.

### Immunohistochemistry

Immunostaining was performed using the avidin-biotin-peroxidase complex method (Ultrasensitive^TM^, MaiXin, Fuzhou, China). Monoclonal goat anti-CK14 and anti-Oct3/4 were purchased from Santa Cruz Biotechnology (Santa Cruz, CA, USA). The experimental protocol was performed according to the manufacturer's instructions. We used the positive control supplied with the kit. Phosphate-buffered saline (PBS) was used in place of the primary antibody as a negative control. The proteins were detected by 3,3′-diaminobenzidine tetrahydrochloride (DAB) staining. Samples were imaged using an Olympus BX50 DIC microscope (Olympus, Shinjuku, Tokyo, Japan).

### Western blotting

Total cell homogenates were prepared by lysing cells in NP40 lysis buffer containing 1% NP40, 10% glycerol, 20 mM Tris-HCl pH 8.0, 137 mM NaCl and 4% complete protease inhibitor cocktail mix (Roche, Mannheim, Germany). Eighty micrograms of total protein was resolved by SDS-PAGE and subsequently transferred onto a PVDF membrane (Immobilon, Millipore, Billerica, MA, USA). Membranes were blocked with 5% non-fat dry milk in PBS for 1 h with gentle shaking. The membranes were then washed three times for 10 min each with PBS containing 0.1% Tween-20 (PBST). The membranes were incubated with the primary antibodies (Santa Cruz; Table 1) diluted in 1% BSA in PBS overnight at 4°C with shaking. The following day, the membranes were washed three times with PBST as described and incubated with the secondary antibodies (Table 1) for 2 h at room temperature. After an additional three washes with PBST, the membranes were incubated with DAB for 2–3 min at room temperature. The blots were subsequently developed in the dark, dried, and photographed. After scanning, densitometric analysis was performed using the IMAGEJ 1.33 software (National Institutes of Health, Bethesda, MD, USA).

**Table 1:** Antibodies used in western blot analyses

Primary antibody				Secondary antibody (Peroxidase-conjugated)		
Name	Source	Dilution	Product no.	Name	Dilution	Product no.
Oct3/4		1: 200	sc-8628			
	Goat			rabbit anti-goat IgG	1: 2000	sc-2768
Sox2		1: 100	sc-17320			
Nanog		1: 100	sc-33760			
	Rabbit			goat anti-rabbit IgG	1: 2000	sc-2004
β-actin		1: 200	sc-7210			

### MSPCR

DNA was extracted from tracheal epithelial cells 0 h, 6 h, and 48 h after 5-FU treatment using an animal tissue genomic DNA Extraction Kit (Beijing, China). The DNA was stored at -20°C after quantitative analysis. The DNA was methylated, modified, and purified using a methylation modification kit (CHEMICON). The final volume of the modified DNA was 50–60 μL. Two pairs of primers were designed to amplify the *Oct3/4*, *Nanog*, and *Sox2* promoters [39–41]. MSPCR primers were designed using the MethPrimer software [42]. All primers are shown in Table 2. MSPCR was performed with the PrimeSTARTM HS DNA Polymerase (Takara Bio, Otsu, Japan) according to the manufacturer's protocol. The MSPCR conditions were as follows: 95°C for 3 min followed by 40 cycles of three steps at 98°C for 10 s, variable temperature (see Table 2) for 10 s, 72°C for 1 min, and 72°C for 7 min. The MSPCR products were visualized by ethidium bromide staining on 2% agarose gels using an ultraviolet gel scanner (UVP, Cambridge, U.K.).

**Table 2:** RT-PCR and MSPCR primer sequences and product sizes

Gene	Forward sequence (5′→ 3′)	Reverse sequence (5′→ 3′)	Size (bp)	Annealing temp. (°C)
*RT-PCR*				
*Oct3/4*	AGGCAGGAGCACGAGTGGA	CGAAGCGGCAGATGGTTGT	264	58.3
*Nanog*	TCTCCTCCGCCTTCCTCT	TTGCCTCTGAAACCTATCCTTG	204	53.1
*Sox2*	GGGCTCTGTGGTCAAGTC	TAGTCGGCATCACGGTTT	435	62.1
*β-actin*	CCAAGGCCAACCGCGAGAAGATGAC	AGGGTACATGGAGCCGCCAGAC	587	58
MSPCR				
*Oct3/4 M*	ATCTGCCCATTGTGGGGAAGTTT	GCTGAGCCTTCATTCCTGCCCT	183	64
*U*	TAATTTGTTTATTGTGGGGAAGTTT	ACTAAACCTTCATTCCTACCCTCTC	185	60.3
*Nanog M*	CTTCTGTGCAGGAGGTGTCTTCCA	CCCTCTAGCTCTTCAGTTGGCTTTTT	191	62.1
*U*	TTTTGTGTAGGAGGTGTTTTTTAGA	CCCTCTAACTCTTCAATTAACTTTTT	190	62
*Sox2 M*	CTCCCACAGCCTGGGCTTGC	CTGCTGATTGGCACAGTGGTAGTC	158	58.7
U	TATTTTTTTATAGTTTGGGTTTGTT	CCCCTACTAATTAACACAATAATAATC	165	58

M: methylation-sensitive.

U: unmethylation-sensitive.

### Measurement of DNA methyltransferase activity (DNMT1, DNMT3a, and DNMT3b)

Nuclear proteins were extracted from the tracheal epithelium of both normal and 5-FU-treated rats. Samples were collected 0 h, 6 h, 24 h, and 48 h after treatment with 5-FU. The extraction of both nuclear and cytosolic proteins was performed with an extraction kit (Bi Yuntian). All steps were performed on ice. The concentration of nuclear protein was estimated using the BCA method. Nuclear protein extracts were added to 96-well, enzyme-labeled plates, and the DNA methyltransferase activity was measured based on absorbance at 510 nm at 37°C. The assay was performed according to the manufacturer's instructions (DNMT Activity/Inhibition Assay Kit, GENMED Scientific, Shanghai, China). The sample activities were converted using the formula below and activity curves generated:

Activity calculation formula:

[(sample reading - background reading) x sample dilution multiple × 0.125 (system capacity; mL)] ÷ [0.005 (sample capacity; mL) × 6.58 (mmol absorption coefficient) × 0.6 (cm) × 15 (min) ] = units/mL ÷ (sample protein concentration) mg/mL = units/mg

Units = μmoles S-adenosylmethionine/min

### Indirect immunofluorescence

Indirect immunofluorescence staining was performed on serial 3-μm thick tracheal tissue sections from both the untreated and 5-azaC-treated groups (Santa Cruz Biotechnology, Santa Cruz, CA, USA). The secondary antibodies were the following: Tetramethylrhodamine isothiocyanate (TRITC)-conjugated rabbit anti-goat immunoglobulin G (and fluorescein isothiocyanate-conjugated goat anti-rabbit IgG (dilution 1:100; HuaMei, Beijing, China). The secondary antibodies were diluted in PBS containing 1% bovine serum albumin (BSA) prior to use. After treatment with the secondary antibody, the samples were incubated with 0.5 μg/mL 4, 6-diamidino-2-phenylindole (DAPI; Sigma, St. Louis, MO, USA) for nuclear counterstaining. The samples were imaged using an epi-illumination fluorescence microscope BX50 (Olympus, Tokyo, Japan).

### RT-PCR analysis

Total RNA was extracted from harvested cells with the TRIzol reagent (Invitrogen, Carlsbad, CA, USA). RT-PCR was performed using the TaKaRa RNA PCR Kit (AMV) version 3.0 (Takara Bio, Otsu, Japan) according to the manufacturer's protocol. PCR primers were designed to span exons in order to minimize the possibility of genomic DNA contamination (Table 2). ß-actin was used as an endogenous control. The PCR conditions were as follows: 94°C for 2 min, 94°C for 30 s, variable temperature (see Table 2) for 40 s, and 72°C for 1 min, for 35 cycles. PCR reactions that lacked reverse transcriptase served as negative controls. The PCR products were visualized by ethidium bromide staining on 2% agarose gels using a gel scanner (UVP, Cambridge, U.K.).

### Statistical analysis

Data from at least three independent experiments were used for statistical analyses. All analyses were performed with the SPSS 11.5 software (SPSS, Chicago, IL, USA). All values are expressed as the mean ± standard deviation. One-way ANOVA was used to evaluate statistical significance, and a *P-value* < 0.05 was considered significant.

## References

[1] Weissman IL (2000). Stem cells: units of development, units of regeneration, and units in evolution. Cell.

[2] Cheung TH, Rando TA (2013). Molecular regulation of stem cell quiescence. Nat Rev Mol Cell Biol.

[3] Hadas RA, Sara B, Virag V, Danna Y, Andras D (2013). Tissue resident stem cells: till death do us part. Biogerontology.

[4] Ghosh M, Helm KM, Smith RW, Giordanengo MS, Li B, Shen H, Reynolds SD (2011). A single cell functions as a tissue-specific stem cell and the *in vitro* niche-forming cell. Am J Respir Cell Mol Biol.

[5] Hegab AE, Ha VL, Gilbert JL, Zhang KX, Malkoski SP, Chon AT, Darmawan DO, Bisht B, Ooi AT, Pellegrini M, Nickerson DW, Gomperts BN (2011). Novel stem/progenitor cell population from murine tracheal submucosal gland ducts with multipotent regenerative potential. Stem Cells.

[6] Hegab AE, Nickerson DW, Ha VL, Darmawan DO, Gomperts BN (2012). Repair and regeneration of tracheal surface epithelium and submucosal glands in a mouse model of hypoxic-ischemic injury. Respirology.

[7] Liras A (2010). Future research and therapeutic applications of human stem cells: general, regulatory, and bioethical aspects. J Transl Med.

[8] Mimeault M, Batra SK (2008). Recent Progress on Tissue-Resident Adult Stem Cell Biology and Their Therapeutic Implications. Stem Cell Rev.

[9] Filip S, Mokry J, Hruska I (2003). Adult stem cells and their importance in cell therapy. Folia Biol. (Prague).

[10] Rosner MH, Vigano MA, Ozato K, Timmons PM, Poirier F, Rigby PW, Staudt LM (1990). A POU-domain transcription factor in early stem cells and germ cells of the mammalian embryo. Nature.

[11] Babaie Y, Herwig R, Greber B, Brink TC, Wruck W, Groth D, Lehrach H, Burdon T, Adjaye J (2007). Analysis of Oct4-dependent transcriptional networks regulating selfrenewal and pluripotency in human embryonic stem cells. Stem Cells.

[12] Kehler J, Tolkunova E, Koschorz B, Pesce M, Gentile L, Boiani M, Lomeli H, Nagy A, McLaughlin KJ, Scholer HR, Tomilin A (2004). Oct4 is required for primordial germ cell survival. EMBO Rep.

[13] Yamagata K, Ueda J, Mizutani E, Saitou M, Wakayama T (2010). Survival and death of epiblast cells during embryonic stem cell derivation revealed by long-term live-cell imaging with an Oct4 reporter system. Dev Biol.

[14] Hatano SY, Tada M, Kimura H, Yamaguchi S, Kono T, Nakano T, Suemori H, Nakatsuji N, Tada T (2005). Pluripotential competence of cells associated with Nanog activity. Mech Dev.

[15] Okumuranakanishi S, Saito M, Niwa H, Ishikawa F (2005). Oct-3/4 and Sox2 Regulate Oct-3/4 Gene in Embryonic Stem Cells. J Biol Chem.

[16] Wang J, Rao S, Chu J, Shen X, Levasseur DN, Theunissen TW, Orkin SH (2006). A protein interaction network for pluripotency of embryonic stem cells. Nature.

[17] Mitsui K, Tokuzawa Y, Itoh H, Segawa K, Murakami M, Takahashi K, Maruyama M, Maeda M, Yamanaka S (2003). The homeoprotein nanog is required for pluripotency in mouse epiblast and ES cells. Cell.

[18] Becskei A, Serrano L (2000). Engineering stability in gene networks by autoregulation. Nature.

[19] Boyer LA, Lee TI, Cole MF, Johnstone SE, Levine SS, Zucker JP, Guenther MG, Kumar RM, Murray HL, Jenner RG, Gifford DK, Melton DA, Jaenisch R, Young RA (2005). Core transcriptional regulatory circuitry in human embryonic stem cells. Cell.

[20] Mason RJ, Williams MC, Moses HL, Mohla S, Berberich MA (1997). Stem cells in lung development, disease, and therapy. Am J Respir Cell Mol Biol.

[21] Rock JR, Onaitis MW, Rawlins EL, Lu Y, Clark CP, Xue Y, Randell SH, Hogan BLM (2009). Basal cells as stem cells of the mouse trachea and human airway epithelium.

[22] Song N, Jia XS, Jia LL, Ma XB, Li F, Wang EH, Li X (2010). Expression and role of Oct3/4, Nanog and Sox2 in regeneration of rat tracheal epithelium. Cell Prolif.

[23] Ma XB, Jia XS, Liu YL, Wang LL, Sun SL, Song N, Wang EH, Li F (2009). Expression and role of Notch signalling in the regeneration of rat tracheal epithelium. Cell Prolif.

[24] Ding Q, Jia XS, Zhou Y (2004). Study of tracheal regeneration after injury induced by 5-fluorouracil in rats. Zhonghua Bing Li Xue Za Zhi.

[25] Li X, Xu JX, Jia XS, Li WY, Han YC, Wang EH, Li F (2016). Dormancy activation mechanism of tracheal stem cells. Oncotarget.

[26] Simonsson S, Gurdon J (2004). DNA demethylation is necessary for the epigenetic reprogramming of somatic cell nuclei. Nat Cell Biol.

[27] Ohgane J, Hattori N, Oda M, Tanaka S, Shiota K (2002). Differentiation of trophoblast lineage is associated with DNA methylation and demethylation. Biochem Biophys Res Commun.

[28] Shiota K, Kogo Y, Ohgane J, Imamura T, Urano A, Nishino K, Tanaka S, Hattori N (2002). Epigenetic marks by DNA methylation specific to stem, germ and somatic cells in mice. Genes Cells.

[29] Tycko B (2000). Epigenetic gene silencing in cancer. J Clin Invest.

[30] Tsuji-Takayama K, Inoue T, Ijiri Y, Otani T, Motoda R, Nakamura S, Orita K (2004). Demethylating agent, 5-azacytidine, reverses differentiation of embryonic stem cells. Biochem Biophys Res Commun.

[31] Zhao XD, Han X, Chew JL, Liu J, Chiu KP, Choo A, Orlov YL, Sung WK, Shahab A, Kuznetsov VA, Bourque G, Oh S, Ruan Y (2007). Wholegenome mapping of histone H3 lys4 and 27 trimethylations reveals distinct genomic compartments in human embryonic stemcells, Cell StemCell.

[32] Pan G, Tian S, Nie J, Yang C, Ruotti V, Wei H, Jonsdottir GA, Stewart R, Thomson JA (2007). Whole-genome analysis of histone H3 lysine 4 and lysine 27 methylation in human embryonic stem cells. Cell Stem Cell.

[33] Guenther MG, Levine SS, Boyer LA, Jaenisch R, Young RA (2007). A chromatin landmark and transcription initiation at most promoters in human cells. Cell.

[34] Bird A (2002). DNA methylation patterns and epigenetic memory. Genes Dev.

[35] Smith ZD, Chan MM, Mikkelsen TS, Gu H, Gnirke A, Regev A, Meissner A (2012). A unique regulatory phase of DNA methylation in the early mammalian embryo. Nature.

[36] Ziller MJ, Gu H, Müller F, Donaghey J, Tsai LT-Y, Kohlbacher O, Jager PLD, Rosen ED, Bennett DA, Bernstein BE, Gnirke A, Meissner A (2013). Charting a dynamic DNA methylation landscape of the human genome. Nature.

[37] Li JY, Pu MT, Hirasawa R, Li BZ, Huang YN, Zeng R, Jing NH, Chen TP, Li E, Sasaki H, Xu GL (2007). Synergistic Function of DNA Methyltransferases Dnmt3a and Dnmt3b in the Methylation of Oct4 and Nanog. Mol Cell Biol.

[38] Shakya A, Callister C, Goren A, Yosef N, Garg N, Khoddami V, Nix D, Regev A, Tantin D (2015). Pluripotency Transcription Factor Oct4 Mediates Stepwise Nucleosome Demethylation and Depletion. Mol Cell Biol.

[39] Wu DY, Yao Z (2005). Isolation and characterization of the murine Nanog gene promoter. Cell Res.

[40] Wiebe MS, Wilder PJ, Kelly D, Rizzino A (2000). Isolation, characterization, and differential expression of the murine Sox-2 promoter. Gene.

[41] Nordhoff V, Hübner K, Bauer A, Orlova I, Malapetsa A, Schöler HR (2001). Comparative analysis of human, bovine, and murine Oct-4 upstream promoter sequences. Mammalian Genome.

[42] Li LC, Dahiya R (2002). MethPrimer: designing primers for methylation PCRs. Bioinformatics.

